# Acetyl-L-Carnitine Supplementation During HCV Therapy With Pegylated Interferon-α 2b Plus Ribavirin: Effect on Work Performance; A Randomized Clinical Trial

**DOI:** 10.5812/hepatmon.11608

**Published:** 2014-05-05

**Authors:** Giulia Malaguarnera, Manuela Pennisi, Caterina Gagliano, Marco Vacante, Michele Malaguarnera, Salvatore Salomone, Filippo Drago, Gaetano Bertino, Filippo Caraci, Giuseppe Nunnari, Mariano Malaguarnera

**Affiliations:** 1 Department of Clinical and Molecular Biomedicine, Section of Pharmacology and Biochemistry, University of Catania, Catania, Italy; 2 Department of Neurosciences, University of Catania, Catania, Italy; 3 The Great Senescence Research Center, University of Catania, Catania, Italy; 4 Department of Internal Medicine and Systemic Diseases, University of Catania, Catania, Italy; 5 Department of Educational Sciences, University of Catania, Catania, Italy; 6 Institute for Research on Mental Retardation and Brain Aging, Troina, Italy; 7 Department of Clinical and Molecular Biomedicine, Division of Infectious Diseases, University of Catania, Catania, Italy

**Keywords:** Acetyl Lcarnitine, Interferon, Ribavirin, Hepatitis C, Fatigue, Quality of Life

## Abstract

**Background:**

The health status of employees with chronic hepatitis C has major implications for organizations and labour market.

**Objectives:**

To assess the effects of Acetyl-L-Carnitine administration on work productivity, daily activity, and fatigue in subjects with chronic hepatitis C treated with Pegylated-Interferon-α2b and Ribavirin.

**Patients and Methods:**

In this prospective, randomized, placebo controlled, double blind clinical trial, 30 subjects (Group A) with chronic hepatitis, received Pegylated-Interferon-α2b (1.5 mg/kg per week) plus Ribavirin and placebo, while 32 subjects (Group B) received the same dosage of Pegylated-Interferon-α2b plus Ribavirin plus 2g Acetyl-L-Carnitine twice per day, for 12 months. Work productivity loss, impairment in daily activities, presenteeism, absenteeism, have been assessed using the Work Productivity and Activity Impairment questionnaire. We also evaluated severity of fatigue, mental fatigue and physical fatigue.

**Results:**

Significant difference were observed in physical fatigue, mental fatigue and severity of fatigue, aspartate aminotransferase, alanine aminotransferase, and viremia after 12 months treatment. In Group B we observed a significant decrease of presenteeism and daily activity impairment after 6 months, 12 months and at follow up. A significant increase of work productivity was observed after 12 months and at follow up.

**Conclusions:**

Office workers with chronic hepatitis C, treated with Pegylated-Interferon-α2b plus Ribavirin, had work performance loss. In subjects treated with Acetyl-L-Carnitine supplementation we observed increased daily activity and reduced presenteeism and fatigue. Acetyl-L-Carnitinegroup had a smaller reduction of productivity comparing to placebo group.

## 1. Background

Hepatitis C virus (HCV) infection is currently a major health problem. HCV affects 4.1 million people in the United States, of whom 3.2 million are estimated to be chronically infected (1). Administration of Pegylated Interferon-a (Peg-INF-a) and Ribavirin (RBV) may reach eradication rates of 75%–80% in patients with HCV genotypes 2 and 3 and between 45% and 52% in patients with genotype 1 (2, 3). HCV infection not only compromises health related quality of life, but has been also associated with social marginalization, impairment of intimate and family relationships, reduced sense of well-being due to fear of transmission and prognosis, fatigue, hopelessness and depression (4, 5). HCV is one of the few infections, in addition to HIV, which is heavily linked to psychiatric disorders (6). The health status of employees with chronic hepatitis C has major implications for organizations and labour market. Acetyl-L-carnitine is a versatile endogenous molecule present in mammalian metabolism. Carnitine is synthesized from the essential aminoacids lysine and methionine in kidney, liver and brain (7). L-carnitine, acetyl-L-carnitine and various carnitine enzymes play a relevant role in cellular energy production (8) being involved in the carriage of long-chain fatty acids into the mitochondrial matrix for beta oxidation (9). Previous studies showed that carnitine and its derivatives reduce depression, both physical and mental fatigue, improves cognitive functions and quality of life (10, 11). In HCV patients treated with IFN alone or with ribavirin we observed that L-carnitine supplementation improves biochemical response and increases hematological pattern (12). In recent years occupational therapies have been developed for various pathologies that focus on quality of life including physical and mental health. Most of these intervention showed some improvement on quality of life or on other physical and psychological outcomes, but did not pay attention to the aspect of work which is considered to be an important contributor to the quality of life.

## 2. Objectives

We assessed the effects of ALC administration on changes in work productivity, daily activity, and fatigue in subjects with chronic hepatitis C treated with Peg-IFN-a and RBV. The primary outcome was the assessment of changes in Work Productivity and Activity Impairment and Fatigue Severity Scale scores; the secondary analysis included evaluation of liver function tests (AST, ALT) and viremia.

## 3. Patients and Methods

**Figure 1.**
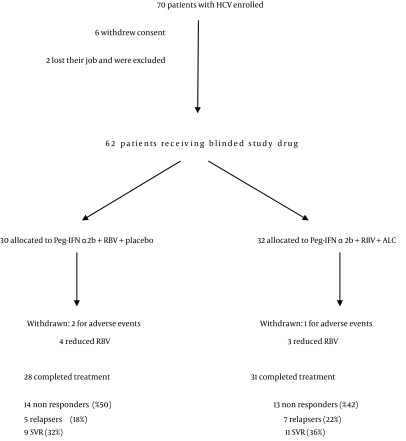
Trial Profile of Peg-IFNα2b Plus RBV Plus ALC Treatment

The study was designed as a prospective, randomized, placebo controlled, double-blind clinical trial. The study was conducted at the Department of Senescence, Cannizzaro Hospital, University of Catania (Italy), between January 2010 and December 2011. 62 patients have been enrolled (36 males, 26 females) (Table 1). The patients received Peg-IFN-a 2b plus ribavirin (group A; n = 30) and placebo or Peg-IFN-a 2b plus ribavirin plus Acetyl-L-Carnitine (group B; n = 32) for a 12-month period (Figure 1). Patients were randomly divided into 2 groups (Group A and Group B) and stratified according to HCV genotype (1 vs. others) and viral load (< 600,000 vs. > 600,000 IU/mL). Patients were randomized into two groups (Acetyl-L-carnitine and placebo) using permuted-block randomization with an allocation ratio of 1:1 and a block size of 4. Randomization was performed by an independent statistician. Random numbers were assigned to patients according to the sequence of their inclusion and patients received respective study products. Both clinical investigators and patients were blind to the product given. Peg-IFN-a 2b (1.5 mg/kg per week) plus RBV and placebo were administered to subjects in Group A. The dose of RBV was 800mg for body weight less than 60 kg, 1,000 mg between 60 and 75 kg, and 1,200 mg more than 75 kg. Subjects in Group B received Peg-IFN-a 2b and RBV plus 2 g ALC administered twice a day per os. Subjects were evaluated before starting therapy, after 6 and 12 months. A follow-up was carried out 6 months after the end of the treatment. Eligible patients were workers who were 18 years of age or older, were infected by HCV and had a quantifiable serum HCV RNA level (as determined by polimerase chain reaction, COBAS AmpliPrep/COBAS TaqMan – ROCHE). HCV infected populations must had elevated serum alanine transaminase levels and findings on liver biopsy consistent with chronic infection. Cirrhotic patients had to have a Child-Pugh score less than 7 to be eligible. Ineligible patients were those who had other liver diseases, as well as those who were affected by cancer, severe jaundice, pulmonary and renal chronic diseases, prostatic diseases, autoimmune diseases and diabetes mellitus. None of the patients made excessive use of alcohol (>20 g/die) or hepato-toxic drugs. Other causes of exclusion included decompensated cirrhosis, pregnancy, and contraindications for Peg-IFN-a or RBV therapy such as cardiopathy, hemoglobinopathies, hemocromatosis, major depression or other severe psychiatric pathological conditions. Clinical evaluations, hematochemical, virological, instrumental and histological analysis were performed on these patients. All subjects underwent a physical examination and medical interview before treatment. Study recruitment was performed in observation and respect of Helsinki Declaration (13). All patients gave their informed consent for the study participation and for each invasive procedure they underwent. All sensitive data were collected and protected in respect of present privacy statements.

**Table 1.** Patients Characteristics at Liver Biopsy ^a^

Parameter	Group A, n = 30, (Peg-IFNα + RBV+ Placebo)	Group B, n = 32, (Peg-IFNα + RBV + ALC)	P Value
**Male**	18 (60)	18 (56)	0.764
**Female**	12 (40)	14 (46)	0.764
**Route of transmission of HCV (No of patients)**			
Blood transfusion	14 (46)	10 (31)	0.211
Intravenous drug abuse	4 (13)	7 (21)	0.378
Occupational	2 (6)	3 (9)	0.696
Unknown	10 (33)	12 (40)	0.727
**HCV genotype**			
1a	1 (3)	2 (6)	0.596
1b	25 (83)	24 (80)	0.417
2a	2 (6)	2 (6)	0.944
3a	2 (6)	4 (12)	0.435
**Blue collars (manual labourers)**	12 (40)	10 (31)	0.471
**White collar (non manual/office labourers)**	18 (60)	22 (68)	0.471
**Fibrosis Score**			
F0	4 (13)	5 (15)	0.794
F1	5 (16)	4 (12)	0.638
F2	13 (43)	10 (31)	0.327
F3	5 (16)	9 (28)	0.280
F4	3 (10)	4 (12)	0.756

^a^ Data are presented in No. %.

### 3.1. Serum Analysis

All patients underwent a complete virological assay for HBV and HCV. HBsAg (Hepatitis B surface Antigen), anti-HBc IgG (Hepatitis B “core” IgG Antibody), HBeAg (Hepatitis B “e” Antigen), HBeAb (Hepatitis B “e” Antibody), HBV-DNA (Hepatitis B Virus DNA), anti-Delta (Delta virus antibody) assays were performed. Anti-HCV antibodies were determined by ELISA (Enzyme-Linked immunosorbent assay ELISA. assay - Ortho Diagnostic Systems, Raritan, NJ, USA). HCV-RNA (Hepatitis C Virus RNA) levels were detected by polymerase chain reaction (PCR) of HCV-RNA 5’UTR using COBAS AmpliPrep/COBAS TaqMan (Roche Diagnostics Systems, Branchburg, N.J). Serum samples negative for HCV RNA were re-tested using a more sensitive standardized qualitative PCR assay with a lower limit of detection of about 100 copies/mL to confirm HCV-RNA disappearance. HCV genotypes and subtypes were identified (14). HCV viral genotypes were determined by restriction analysis of HCV-RNA 5’ UTR (15). AST and ALT (Aspartate Aminotransferase and Alanine Aminotransferase), γGT (gamma GlutamilTranferase), total, conjugated and unconjugated bilirubin, serum proteins analysis were performed. Genetic testing to identify hemochromatosis HFE gene mutations was performed in order to exclude subjects with hereditary hemochromatosis. All liver function tests, hematochemical measurements and virological analysis have been executed in the laboratory of our hospital with automated and standardized methods, in conformity to the quality certified standards EN ISO 9001:2000.

### 3.2. Histological Grading Assessment

Patients underwent ultrasound-assisted percutaneous biopsy: tissue specimens were obtained with Menghini modified needles (Automatic Aspiration Needle for Liver Biopsy, ACR 16G, 11 cm, manufactured by Sterylab Srl, Milan-Italy). A biopsy was considered adequate for evaluation if the specimen was > 1.5 cm long and contained a minimum of 6 portal tracts. Knodell and Ishak Histological activity index (HAI) score was used to assess the histological grading of the disease (16). The METAVIR scoring system was used to stage liver fibrosis as follows: F0, no fibrosis; F1, portal fibrosis without septa; F2, portal fibrosis and few septa; F3, numerous septa without cirrhosis; F4, cirrhosis (17).

### 3.3. Assessment of Fatigue

Wessely and Powell’s test was used to evaluate mental fatigue (total score: 0–10) and physical fatigue (total score: 0–16) (18). Severity of fatigue was evaluated by a self-assessed questionnaire (Fatigue severity scale - FSS) [score range: 1 (no signs of fatigue) to 7 (most disabling fatigue); total score: 9-63] (19).

### 3.4. WPAI (Work Productivity and Activity Impairment)

WPAI is a self-administered questionnaire that was used to evaluate work productivity (20, 21). Subjects were asked information about the number of total working hours, the hours lost due to HCV treatment, the effects on productivity during the past week. We obtained scores for work productivity loss, absenteeism, presenteeism, and impairment in regular daily activities: Absenteeism = hours missed/hours missed + hours worked; Presenteeism = scale score/10; Work productivity loss = absenteeism + (hours worked x presenteeism); Daily activity impairment = scale score/10. The results were converted to percentages. FSS and WPAI questionnaires were not validated for Italian population.

### 3.5. Efficacy and Safety Assessment

We performed an intention-to-treat (ITT) efficacy analysis. ‘‘Sustained virological responders’’ (SVR) were patients with not identifiable serum HCV RNA at the end of the study. We considered the “relapse” as undetectable HCV-RNA levels at the end of treatment but detectable levels during the follow-up period. Reasons for discontinuation of the treatment were severe adverse events and absence of compliance.

### 3.6. Statistical Analysis

Results are expressed as means ± standard deviations. Quantitative data were compared by paired or unpaired Student’s t-test or Mann–Whitney test; the χ-square test was used for analysis of qualitative data. Considering the study power of 90%, type one error of 5%, with respect to 20% ofdropouts, the sample size for each group was determinedto be 30 (9). All results shown in this manuscript were analyzed in the intention-to-treat population. P values < 0.05 were considered statistically significant. All statistical analysis were performed using SPSS 15.0 (Chicago, IL).

## 4. Results

Demographics characteristics were analogous between the two groups at baseline. The most frequent viral genotype was 1b (Table 1). 28 subjects in Group A and 31 subjects in Group B completed the treatment. The non responders were respectively 14 and 13 (50% vs. 42%), the relapsers were 5 and 7 (18% vs. 22%) and the SVR were 9 and 11 (32% vs. 36%) (Figure 1).

### 4.1. Effect of ALC on Work Productivity and Fatigue

In Group A physical fatigue decreased after 6 months (P = 0.0194). No significant differences were observed as regards absenteeism, presenteeism, work productivity loss and daily activity impairment (Table 3). In Group B after 6-12 months and at follow up we observed a significant decrease in physical fatigue (P < 0.0001), mental fatigue (P < 0.0001), and FSS (P = 0.0006). We observed a significant decrease of presenteeism after 6 months (P = 0.0458), 12 months (P = 0.0159) and at follow up (P = 0.0257). Daily activity impairment was also decreased after 6 months (P = 0.0240), 12 months (P = 0.0031) and at follow up (P = 0.0186). A significant increase of work productivity was observed after 12 months (P = 0.0112) and at follow up (P = 0.0298). The comparison between Group A and Group B showed a significant difference after 6 months in physical fatigue (- 1.4 versus -3.3; P = 0.0014) and FSS (- 1.8 versus - 8.3; P = 0.0075). After 12 months, we observed a significant difference in physical fatigue (-1.2 versus -7.0; P < 0.0001), mental fatigue (-0.4 versus -2.2; P < 0.0001) and FSS (–4.0 versus -15.0; P < 0.0001). At follow up, there were significant differences in physical fatigue (-1.0 versus -4.1; P < 0.0001) and FSS (-0.3 versus –11.3; P < 0.0001).

### 4.2. Effect of ALC on Transaminases, Viremia and HAI

In Group A, there was a significant decrease in AST (P < 0.0001) and ALT (P < 0.0001) after 6-12 months and at follow up. Viremia was significantly reduced after 6 months (P = 0.0097), 12 months (P = 0.0007), and at follow up (P = 0.0012) (Table 2). HAI score decreased after 12 months (P = 0.0338). In the group that was treated with Peg-IFNα plus RBV plus ALC we observed a significant decrease in AST (P < 0.0001) and ALT (P < 0.0001), after 6-12 months, and at follow up. A significant decrease in HAI score (P = 0.0005) was observed after 12 months. A decrease in viremia was observed after 6 months (P = 0.0007), 12 months (P < 0.0001), and at follow up (P < 0.0001). The comparison between group A and group B showed a significant difference after 12 months in AST (-123.2 versus -93.1; P < 0.0001), ALT (-138.1 versus –98.4; P < 0.0001), and viremia (-3.2 versus –2.2; P = 0.0047). At follow up, we observed a significant difference in AST (-110.4 versus –89.7; P = 0.0013). Adverse events observed in both groups are reported in Table 4.

**Table 2.** Patients Characteristics at Liver Biopsy, Values Are Expressed as Mean (SD)

Parameter	Group A, n = 30, (Peg-IFNα + RBV + Placebo)	Group B, n = 32, (Peg-IFNα + RBV + ALC)	P Value
**Mean age, y**	44.9 (4.2)	48.6 (3.8)	0.0006
**HCV exposure time, y**	6.44 (4.8)	6.35 (4.9)	0.942
**BMI, kg/m** ^**2**^	26.8 (4.2)	26.4 (4.8)	0.728
**Plasma glucose, (mmol/l) (normal 3.9-6.4)**	5.81 (0.86)	5.77 (0.84)	0.853
**AST, IU/l, (normal 15-50)**	171 (39.1)	185.4 (38.2)	0.147
**ALT, IU/l, (normal 15-50)**	190.4 (39.8)	188.8 (37.9)	0.871
**Viremia, 10** ^**6**^ ** copies/ml**	5.11 (2.46)	5.16 (2.84)	0.941
**CRP, mg/dL, (normal <1.0)**	4.9 (0.5)	5.1 (0.61)	0.164
**HAI**	10.6 (3.4)	10.8 (3.4)	0.817

**Table 3.** Characteristics of Subjects at Baseline, After 12 Months, and at Follow-up, Values Are Expressed as Mean (SD) ^a^

	Before Treatment	After 6 Months	P-Value 1^b^	Diff	P Value 2^c^	After 12 Months	PValue ^b^	Diff	PValue ^c^	Follow-up	PValue ^b^	Diff	Pvalue ^c^
**AST, IU/l**	154 (41.2)	87.2 (38.1)	< 0.0001	66.800	0.0044	61.8 (24.1)	< 0.0001	92.200	< 0.0001	65.2 (24.7)	< 0.0001	88.800	0.0013
**ALT, IU/l**	175.2 (41.8)	112.4 (40.7)	< 0.0001	62.800	0.0004	77.2 (15.8)	< 0.0001	98.000	< 0.0001	74.2 (20.2)	< 0.0001	101.000	< 0.0001
**Bilirubin, mmol/l**	10.7 (8.7)	10.8 (7.1)	0.9613	-0.100	0.6846	10.6 (6.9)	0.9608	0.100	0.7510	10.9 (6.8)	0.9213	-0.200	0.6167
**Albumin, g/dl**	4.2 (0.9)	4.1 (0.8)	0.6509	0.100	1.0000	4.1 (0.9)	0.6685	0.100	0.5698	4.2 (0.8)	1.0000	0.000	1.0000
**Viremia, 10** ^**6**^ ** copies/ml**	5.16 (3.08)	3.25 (2.41)	0.0097	1.9100	0.2187	2.88 (1.67)	0.0007	2.2800	0.0047	2.96 (1.72)	0.0012	2.2000	0.0063
**HAI**	10.6 (3.4)	-	-	-	-	8.8 (3.0)	0.0338	1.800	0.3129	-	-	-	-

^a^ Group A Peg-IFN α + RBV + Placebo (n = 30).

^b^ Test mean during time with use of pair sample t test.

^c^ Test mean between differences of two group with use of Students t-test.

**Table 4.** WPAI and Fatigue Scores in the Study Population, Values Are Expressed as Mean (SD)

Group B Peg IFN α + RBV + ALC (n = 32)													
	Before Treatment	After 6 Months	P Value ^a^	Diff	P Value ^b^	After 12 Months	P Value ^a^	Diff	P Value ^b^	Follow-up	P Value^ a^	Diff	P Value ^b^
**Absenteeism**	5.4 (10.8)	3.2 (11.4)	0.4311	2.200	0.5634	3.0 (10.8)	0.3775	2.400	0.6022	4.1 (10.6)	0.6287	1.300	0.9697
**Presenteeism**	38.4 (21.2)	27.8 (20.4)	0.0458	10.600	0.6582	25.6 (20.1)	0.0159	12.800	0.4183	26.4 (20.8)	0.0257	12.000	0.4912
**Work productivity loss**	40.1 (23.4)	29.1 (24.1)	0.0687	11.000	0.8269	26.4 (18.2)	0.0112	13.700	0.5099	28.2 (19.2)	0.0298	11.900	0.4670
**Daily activityimpairment**	45.4 (24.2)	31.8 (22.8)	0.0240	13.600	0.6744	27.8 (21.4)	0.0031	17.600	0.2439	31.4 (22.1)	0.0186	14.000	0.3931
**Physical fatigue**	12.0 (2.1)	8.7 (1.9)	< 0.0001	3.300	0.0014	5.0 (2.4)	< 0.0001	7.000	< 0.0001	7.9 (2.3)	< 0.0001	4.100	< 0.0001
**Mental fatigue**	6.4 (1.8)	4.7 (1.4)	< 0.0001	1.700	0.0175	4.21 (1.2)	< 0.0001	2.1900	< 0.0001	4.6 (1.3)	< 0.0001	1.800	0.0013
**Fatigue severity scale**	49.1 (9.6)	40.8 (8.7)	0.0006	8.300	0.0075	34.1 (8.4)	0.0006	15.000	< 0.0001	37.8 (9.2)	0.0006	11.300	
**Group A Peg-IFN α + RBV + Placebo (n = 30)**													
**Absenteeism**	5.0 (9.4)	4.8 (10.2)	0.9373	0.200	0.5634	4.4 (10.2)	0.8136	0.600	0.6022	4.0 (10.0)	0.6913	1.000	0.9697
**Presenteeism**	32.2 (20.8)	30.2 (22.1)	0.7194	2.000	0.6582	30.0 (22.4)	0.6949	2.200	0.4183	30.2 (22.4)	0.7214	2.000	0.4912
**Work productivity loss**	38.2 (21.8)	30.4 (22.4)	0.1770	7.800	0.8269	29.8 (22.1)	0.1437	8.400	0.5099	32.1 (22.7)	0.2928	6.100	0.4670
**Daily activity impairment**	41.8 (20.9)	34.2 (21.9)	0.1744	7.600	0.6744	34.1 (20.7)	0.1570	7.700	0.2439	36.2 (21.8)	0.3140	5.600	0.3931
**Physical fatigue**	11.8 (2.4)	10.4 (2.1)	0.0194	1.400	0.0014	10.6 (2.4)	0.0577	1.200	< 0.0001	10.8 (2.3)	0.1048	1.000	< 0.0001
**Mental fatigue**	6.1 (1.7)	5.6 (1.5)	0.2320	0.500	0.0175	5.7 (1.4)	0.3240	0.400	< 0.0001	5.8 (1.5)	0.4715	0.300	0.0013
**Fatigue severity scale**	48.2 (8.4)	46.4 (7.1)	0.3738	1.800	0.0075	44.2 (8.7)	0.0752	4.000	< 0.0001	47.9 (8.7)	0.8924	0.300	< 0.0001

^a^ Test mean during time with use of pair sample t test.

^b^ Test mean between differences of two group with use of Students t-test.

**Table 5.** Adverse Events Observed in the Study Population ^a^

	Group A (n = 30) (Peg-IFNα + RBV + placebo)	Group B (n = 32) (Peg-IFNα + RBV + ALC)	P Value
**Psychological disorders**	8	3	0.075
**Hypercholesterolemia **	18	34	0.000
**Fatigue**	55	46	0.000
**Headache**	47	41	0.000
**Musculoskeletal pain**	48	36	0.000
**Myalgia**	55	31	0.000
**Hypertriglyceridemia**	36	28	0.000
**Nausea**	25	18	0.020
**Anorexia**	14	11	0.322
**Irritability**	24	18	0.045
**Hyperglycemia**	12	6	0.065
**Weight loss**	11	6	0.114

^a^ Data are presented as %.

## 5. Discussion

HCV and IFN-treatment have shown elevated risk factors for adverse psychosocial health outcomes from work-place stress/conditions. In HCV patients treated with IFN we observed an increase in depressed or irritable mood, feelings of overwhelming sadness or seeming inability to feel emotions; a marked decrease of interest in activities and hobbies that are normally enjoyed, friends, socializing and a loss of libido; a decreased ability to focus, concentrate or make decisions, memory loss; an increase of mental and physical fatigue severity; a decreased level of energy and changes in activity levels (22-25). As far as we know, few studies have been carried out to date to show a relationship between work and HCV therapy. In Group A we observed a not significant decline of work productivity and activity impairment. In Group B we observed a significant decrease of presenteeism (P < 0.05) and daily activity impairment (P < 0.05) after 6 months and a decrease of presenteeism, work productivity, and daily activity impairment after 12 months (P < 0.01). Fatigue is the most common extra hepatic manifestation of HCV infection with a prevalence of 50% (26-28). IFN-induced fatigue in HCV–infected individuals is associated with some molecular signatures of inflammatory pathways. HCV diagnosis was found to have deteriorating effects on social functioning in most of the studies. In addition to prolonged physical or mental fatigue, qualitative research has also identifiers personal problems such as cognitive limitations, difficulty mobilizing support, difficulty coping with a new self image and changed attitude to work. The treatment with pegylated interferon-α plus ribavirin impairs work performance among office workers, which may lead to a substantial loss to work productivity. The disease burden and its treatment have an indirect cost, such as low employment, absence from work and impaired productivity, and a decrease in quality of life (29, 30). Loss of work productivity may occur through either absenteeism (absence, early leaving) or presenteeism. Presenteeism describes productivity loss when employees come to work but are not fully productive. In white collar workers, scarce accuracy represents the most important modifications in mental functions. The changes affect the whole human processing information system (e.g. the sensoriperceptive system, the cognitive system and the motor system). IFN-alpha induced acute confusional states presenting with psychomotor retardation, disorientation, parkinsonism and psychosis. In HCV patients, an improvement in work capability could reflect higher work productivity as well as better well being and quality of life. The limitations of this study include the small number of patients. Even though we observed an improvement in work performance in both white and blue collars, a larger study population should be considered to obtain more detailed information. It was difficult to evaluate whether the improvement in work performances was due to ALC supplementation or to the therapy with interferon and ribavirin. In previous studies we demonstrated that supplementation with L-carnitine and its derivatives increase the sustained virological response in patients treated with interferon and ribavirin (12). In fact, in sustained virological responders, absenteeism, presenteeism, work productivity loss and daily activity impairment are decreased compared with non responders. Treatment with Peg-IFN and ribavirin led to a reduction in work productivity and to an increase in absenteeism and presenteeism after the 1 month therapy and subsequently after 6 months. Effects of Peg-IFN and ribavirin decrease over time. In HCV patients, an improvement in work capability could reflect higher work productivity as well as better well being and quality of life.Future studies are needed to explore these associations more deeply and develop new approaches to diminish their consequences at the workplace.
